# Early post-operative serum albumin level predicts survival after curative nephrectomy for kidney cancer: a retrospective study

**DOI:** 10.1186/s12894-018-0427-3

**Published:** 2018-12-06

**Authors:** Yongquan Tang, Zhihong Liu, Jiayu Liang, Ruochen Zhang, Kan Wu, Zijun Zou, Chuan Zhou, Fuxun Zhang, Yiping Lu

**Affiliations:** 1 Department of Pediatric Surgery, West China Hospital, Sichuan University, No. 37 of Guoxue Xiang, Chengdu, 610041 China; 2 Department of Urology, Institute of Urology, West China Hospital, Sichuan University, Chengdu, China; 3 Department of Urology, Fujian Provincial Hospital, Fuzhou, China; 4 Department of Urology, the Third Affiliated Hospital, Sun Yat-sen University, Guangzhou, China

**Keywords:** Kidney cancer, Survival, Hypoalbuminemia, Radical resection

## Abstract

**Background:**

Previous studies have shown that albumin-related systemic inflammation is associated with the long-term prognosis of cancer, but the clinical significance of an early (≤ 7 days) post-operative serum albumin level has not been well-documented as a prognostic factor in patients with renal cell cancer.

**Methods:**

We retrospectively included patients hospitalized for kidney cancer from January 2009 to May 2014. First, the receiver operating characteristic analysis was used to define the best cut-off of an early post-operative serum albumin level in determining the prognosis, from which survival analysis was performed.

**Results:**

A total of 329 patients were included. The median duration of follow-up was 54.8 months. Patients with an early post-operative serum albumin level < 32 g/L had a significantly shorter median recurrence-free survival (RFS; 49.1 versus 56.5 months, *P* = 0.001) and median overall survival (OS; 52.2 versus 57.0 months, *P* = 0.049) than patients with an early post-operative serum albumin level ≥ 32 g/L. After adjusting for age, BMI, tumor stage, post-operative hemoglobin concentration, and pre-operative albumin, globulin, and hemoglobin levels, multivariate Cox regression showed that an early post-operative serum albumin level < 32 g/L was an independent prognostic factor associated with a decreased RFS (HR = 3.60; 95% CI,1.05–12.42 [months], *P* = 0.042) and decreased OS (HR = 9.95; 95% CI, 1.81–54.80 [months], *P* = 0.008).

**Conclusion:**

An early post-operative serum albumin level < 32 g/L is an independent prognostic factor leading to an unfavorable RFS and OS. Prospective trials and further studies involving additional patients are warranted.

## Background

Kidney cancer is one of the most common malignancies involving the urogenital system [1]. The most important treatment for kidney cancer is surgical resection, but post-operative recurrences are common, especially for stage II and above. A number of risk factors have been reported; however, prognostication remains difficult [2, 3]. Notably, new prognostic factors have been described in recent years [4–7].

Several studies have reported that the early post-operative neutrophil-to-lymphocyte ratio may be a long-term prognostic factor for some cancers, including pancreatic, prostate, and bladder cancers [8–10]. This association may result from the potential anti-tumor effect of acute inflammation. Albumin is commonly used to evaluate nutritional status, but a recent study has shown that albumin is also involved in the inflammatory/stress reaction [11]. McMillan and colleagues [12] reported that the serum albumin level was positively correlated with the C-reactive protein level in 40 patients with lung or gastrointestinal cancer. Bozzetti and colleagues [13] first reported the phenomenon of frequent hypoalbuminemia status in the early period after extensive surgery. Based on our previous data, the serum albumin level was < 35 g/L in > 50% of patients following curative nephrectomy (*P* < 0.05), then always recovered to the pre-operative level within 2 weeks. Some researchers have concluded that early post-operative hypoalbuminemia is associated with the pre-operative serum albumin level, age, and extent of surgery [12], while other studies have shown that post-operative hypoalbuminemia may lead to unfavorable short-term prognoses, such as acute kidney injury [13, 14], unbalanced substance metabolism [15–17], and surgical site infections [18, 19]. Such findings suggest that albumin has a role in immune-inflammatory reactions [11, 20].

Recent studies have revealed that albumin-related systemic inflammation is associated with the long-term prognosis in patients with advanced gastrointestinal cancer [11, 21, 22]. Cai and colleagues [23] found that hypoalbuminemia 3–5 weeks after initiation of tyrosine kinase inhibitors is independently associated with a significantly decreased progression-free survival (PFS) and overall survival (OS) in patients with advanced kidney cancer. Furthermore, when combined with the Memorial Sloan-Kettering Cancer Center (MSKCC) risk model, hypoalbuminemia improves the efficiency in predicting recurrence-free survival (RFS) and OS [23]. The current retrospective study determined the potential association between early (≤ 7 days) post-operative hypoalbuminemia with long-term prognosis after resection of kidney cancer.

## Methods

### Study population

In this retrospective single-center study we reviewed the electronic medical records of inpatients with kidney cancer who were admitted to the Department of Urology at West China Hospital of Sichuan University (Sichuan, China) between January 2009 and May 2014. All of the patients underwent curative surgery. The inclusion criteria included the following: > 18 years of age; a pathologic diagnosis of kidney cancer; and negative surgical margins. Negative surgical margin was defined as macroscopic evidence on surgical report or microscopic evidence on histopathologic report. The exclusion criteria were as follows: incomplete resection; history of other life-threatening diseases within 5 years before or after surgery; adjuvant or neoadjuvant treatment; and distant metastasis, with the exception of the adrenal gland. The study conformed to the Declaration of Helsinki and was approved by the Ethics Committee of West China Hospital.

### Objectives

We primarily assessed the prognostic value of early post-operative hypoalbuminemia with respect to RFS and OS in the curative resection of kidney cancer. We then determined the optimal cut-off point of the early post-operative serum albumin level in predicting the prognosis of kidney cancer. RFS was defined as the date of surgery to the date of recurrence, and OS was measured from the date of surgery to the date of death. For patients without a recurrence or who did not die, survival was censored at the date of the last follow-up evaluation. To distinguish confounding factors, subgroup analyses were performed.

### Data collection

All data were obtained from medical records. The follow-up project adhered to the National Comprehensive Cancer Network (NCCN) clinical practice guidelines for kidney cancer [24]. Data were collected by two well-trained researchers. Any discrepancies in data interpretation were resolved by consensus of all authors. The data collected included demographic characteristics, date and type of surgery, clinical-pathologic TNM stage at the time of surgery, histopathologic characteristics, and date of recurrence and/or death if available or date of the last follow-up. TNM stage referred to the tumor size (T), local lymph node involvement (N), and remote metastasis (M), and was assessed according to imaging studies, surgical records, and histopathologic reports. TNM stage and anatomic stage/prognostic groups were also guided by the 2018 NCCN guidelines for kidney cancer [24]. Cancer recurrence was defined as unequivocal radiologic or biopsy evidence of emerging local or distant tumor lesions. In addition, we collated serum albumin, globulin, and hemoglobin data obtained pre- and post-operatively.

### Statistics

All statistical analyses were carried out using Stata 14.0 (Stata Corp, College Station, TX, USA). The receiver operating characteristic (ROC) curve analysis was performed to determine the optimal cut-off point for the post-operative serum albumin level for use in the post-operative prognosis. Based on the cut-off point, we divided the patients into two groups, then survival curves were created using the Kaplan-Meier method and compared using the log-rank test. Gender, age, body mass index (BMI), stage, type of surgery, and pathologic pattern for both groups of patients were compared and tested one-by-one. Four-fold or R × C table data were analyzed with a chi-square test. The median of data with a non-normal distribution was designated as the average and analyzed with the Wilcoxon rank-sum test. The mean of data with a normal distribution was designated as the average and analyzed with a t-test [25]. A factor was entered into multivariate Cox regression analysis if a statistical difference existed in both groups of patients. The hazard ratio (HR) was adopted as the measurement. A *P* value < 0.05 indicated statistical significance.

## Results

### Patients and disease characteristics

**Fig. 1:**
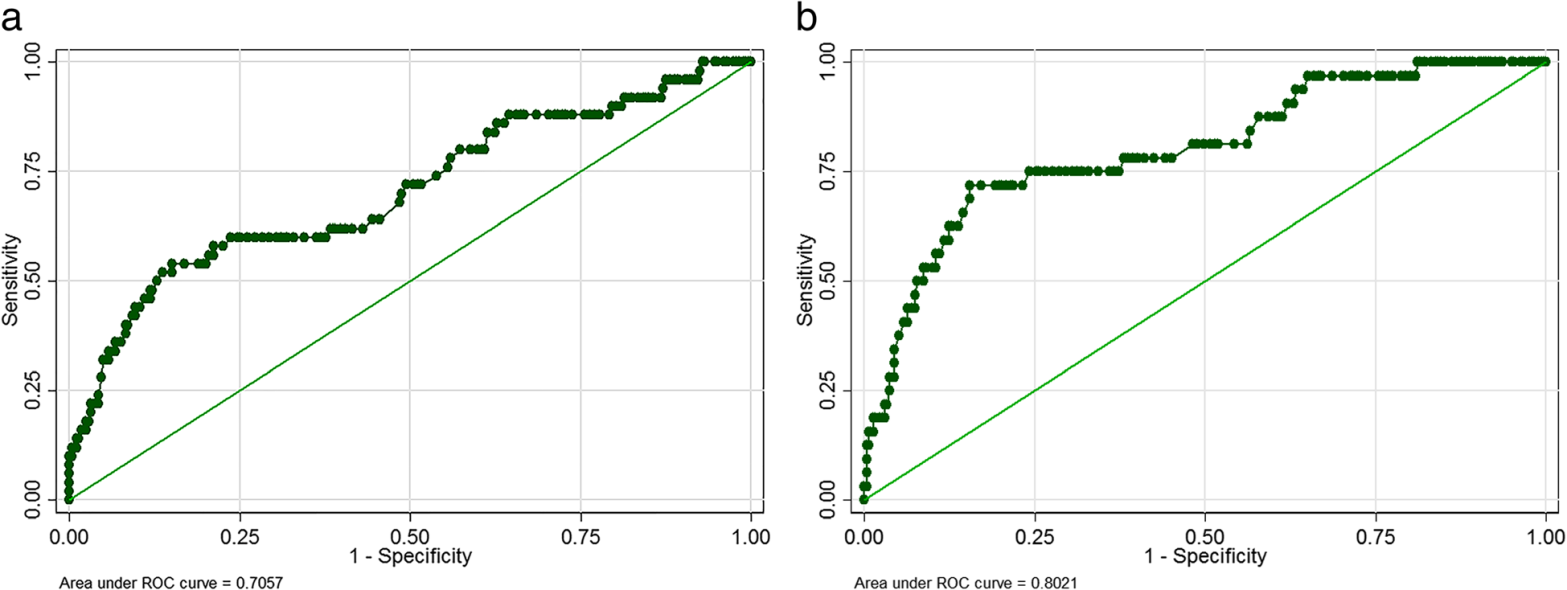
Receiver operating characteristic (ROC) analysis to predict the best cutoff point of early post-operative serum albumin level in predicting recurrence (**a**) and death (**b**) and to calculate the area under the curve (AUC)

A total of 694 patients were available in the database; 329 patients met the inclusion criteria. Among the 329 patients, 64% were male and the median age at the time of surgery was 56 years (range, 22–84 years). The median duration of follow-up was 54.8 months (range, 5.2–96.4 months). The mean pre- and post-operative serum albumin levels were 42.2 and 34.1 g/L, respectively (*P* = 0.000). ROC curve analyses showed that the optimal post-operative serum albumin cut-off level was 32 g/L (area under the curve [AUC] = 0.71) in predicting tumor recurrence (Fig. 1a) and 31 g/L (AUC = 0.80) in predicting death (Fig. 1b). Thus, we used 32 g/L as the cut-off point for grouping comparisons. A total of 99 patients had a serum albumin level < 32 g/L and 230 patients had a serum albumin level ≥ 32 g/L. The patient characteristics and laboratory test results for both groups are presented in Table 1. Age, BMI, tumor stage, post-operative hemoglobin concentration, and pre-operative albumin, globulin, and hemoglobin levels were statistically different (Table 1).

**Table 1:** The baseline and clinic-pathologic characteristics of patients grouped by post-operative serum albumin level

Variable	Serum albumin < 32 g/L (*n* = 99)	Serum albumin ≥ 32 g/L (*n* = 230)	*P*
Gender (%)			0.229
Male	59 (60)	153 (67)	
Female	40 (40)	77 (33)	
Age, y, median(range)	63 (22–82)	53 (24–84)	0.000*
BMI, Kg/m^2^, mean ± SD	22.19 ± 0.38	23.84 ± 0.37	0.007*
Symptom (none/local/system)	(90/9/0)	(211/19/0)	0.805
ECOG-PS (0/1/2/3/4/5)	88/11/0/0/0	213/17/0/0/0	0.267
Stage (%)			0.000*
I	46 (46)	192 (83)	
II	18 (18)	13 (6)	
III	29 (29)	21 (9)	
IV	6 (6)	4 (2)	
Pathological type (%)			0.208
ccRCC	88 (89)	214 (93)	
nccRCC	11 (11)	16 (7)	
Type of surgery (%)			0.149
Radical nephrectomy	73 (74)	151 (66)	
Partial nephrectomy	26 (26)	79 (34)	
Fuhrman grade (%)			0.074
1–2	52 (53)	145 (63)	
3–4	47 (47)	85 (37)	
Preoperative, mean ± SD			
Alb, g/L	39.42 ± 0.43	43.43 ± 0.20	0.000*
Glb, g/L	29.20 ± 0.58	26.09 ± 0.25	0.000*
Hgb, g/L	128.09 ± 2.12	138.24 ± 1.06	0.000*
Post-operative, mean ± SD			
Alb, g/L	36.43 ± 0.19	28.77 ± 0.34	0.000*
Glb, g/L	24.27 ± 0.54	24.16 ± 0.24	0.834
Hgb, g/L	106.81 ± 1.78	123.71 ± 1.15	0.000*

*Significant

*BMI* body mass index, *SD* standard deviation, *ECOP-PS* Eastern Cooperative Oncology Group-performance status, *ccRCC* clear cell renal cell carcinoma, *nccRCC* non-clear cell renal cell carcinoma, *Alb* albumin, *Glb* globulin, *Hgb* hemoglobin

### Survival analysis

**Fig. 2:**
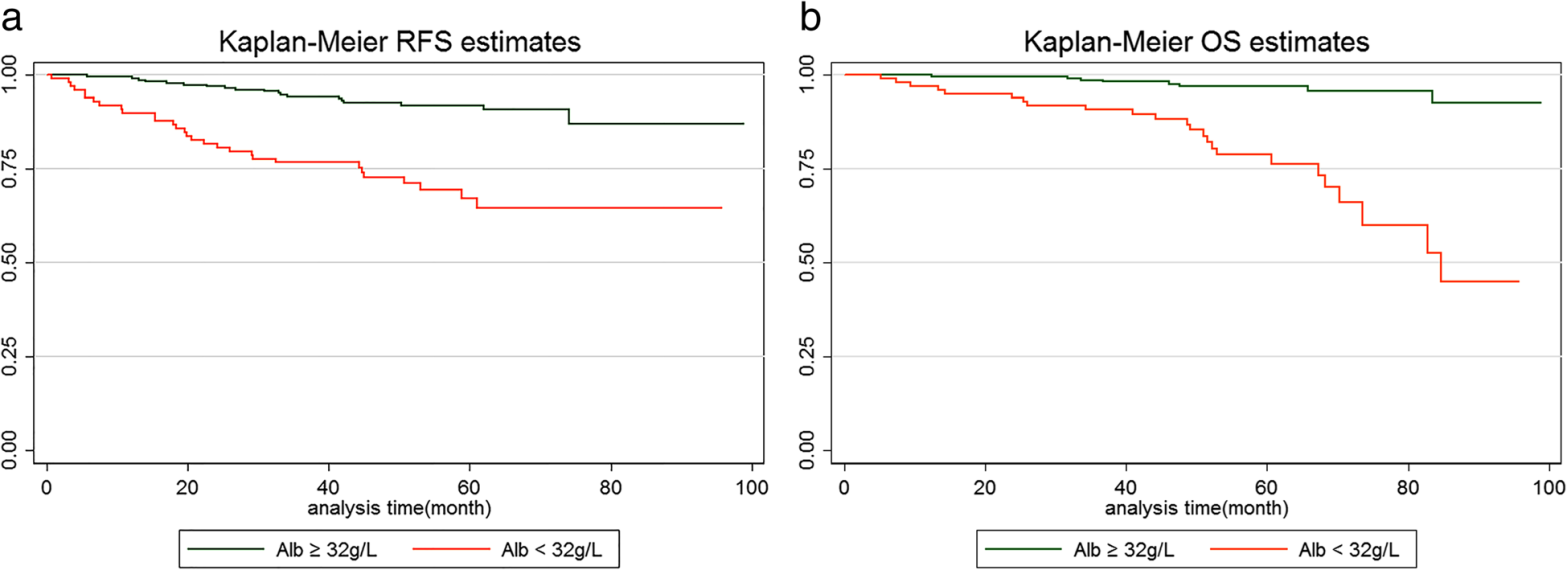
Recurrence-free survival (RFS) (**a**) and overall survival (OS) (**b**) curves of patients with early post-operative serum albumin level ≥32 g/L versus < 32 g/L

No patients were lost to follow-up. The mean duration of follow-up in both groups was similar [58.5 (albumin < 32 g/L) and 59.9 (albumin ≥32 g/L) months, respectively; *P* > 0.05]. During follow-up, 30 patients (30.3%) had tumor recurrences and 24 patients (24.2%) did not survive in the group of patients with a post-operative serum albumin level < 32 g/L. Only 20 patients (8.7%) had tumor recurrences and 8 patients (3.5%) did not survive in the group of patients with a post-operative serum albumin level ≥ 32 g/L. The median RFS of patients with a post-operative serum albumin level < 32 g/L was significantly less than patients with a post-operative serum albumin level ≥ 32 g/L (49.1 and 56.5 months, respectively; *P* = 0.001). The median OS of patients with a post-operative serum albumin level < 32 g/L was also significantly less than patients with a post-operative serum albumin level ≥ 32 g/L (52.2 and 57.0 months, respectively; *P* = 0.049). The group survival curves are shown in Fig. 2. The log-rank test revealed that the differences in group survival curves was significant (*P* = 0.000) for RFS (Fig. 2a) and OS (Fig. 2b).

### Multivariate cox regression

Based on difference testing, age, BMI, tumor stage, post-operative hemoglobin concentration, and pre-operative albumin, globulin, and hemoglobin levels were entered into multivariate Cox regression analysis. An early post-operative serum albumin level < 32 g/L was shown to have an independent impact on the decreased RFS (HR = 3.60; 95% CI,1.05–12.42 [months]; *P* = 0.042) and OS (HR = 9.95; 95% CI, 1.81–54.80 [months]; *P* = 0.008). In addition, tumor stage was also an independent prognostic factor (Table 2). Therefore, subgroup analysis was performed based on tumor stage.

**Table 2:** Effect of early post-operative plasma albumin level on recurrence-free survival and overall survival in multivariate Cox regression analyses adjusted by gender, age, body mass index (BMI), tumor stage and some other laboratory indexes

	Recurrence-free survival			Overall survival		
	HR	95% CI	*P*	HR	95% CI	*P*
Age	0.99	0.95–1.03	0.573	1.00	0.96–1.04	0.980
BMI	0.99	0.88–1.12	0.892	0.97	0.85–1.11	0.684
Stage	1.51	1.03–2.21	0.034*	1.40	0.90-2.17	0.136
Preoperative						
Alb	0.99	0.86–1.13	0.866	0.99	0.83–1.18	0.906
Glb	1.09	0.99–1.19	0.055	1.08	0.99–1.19	0.094
Hgb	1.01	0.98–1.05	0.420	1.00	0.96–1.05	0.816
Post-operative						
Alb < 32 g/L	3.60	1.05–12.42	0.042*	9.95	1.81-54.80	0.008*
Hgb	1.00	0.97-1.04	0.966	1.02	0.98–1.06	0.418

*Significant

*HR* hazard ratio, *CI* confidence interval, *Alb* albumin, *Glb* globulin, *Hgb* hemoglobin

### Sub-group analysis of stages II and III kidney cancer

The survival curves of patients grouped by tumor stage and relevant log-rank testing showed significant differences between stages I and II, and stages III and IV for RFS and OS, but not between stages II and III. Furthermore, after excluding patients with stages I and IV, the two groups had similar TNM stage distributions (*P* = 0.995). Survival analysis and log-rank testing showed that patients with a post-operative serum albumin level < 32 g/L had a significantly decreased RFS (*P* = 0.036) and decreased OS (*P* = 0.012) compared to patients with a post-operative serum albumin level ≥ 32 g/L. Multivariate Cox regression analysis also showed that a early post-operative serum albumin level < 32 g/L was an independent prognostic factor associated with decreased RFS (HR = 6.76; 95% CI, 1.07–42.60; *P* = 0.042) and OS (HR = 26.92; 95% CI, 1.52–477.30; *P* = 0.025).

### Sub-group analysis based on other factors

The histopathologic type of RCC is an important prognostic factor. Clear cell renal cell carcinoma (ccRCC) is the most common histopathologic type of kidney cancer. There were 88 (89%) and 214 (93%) patients in the post-operative serum albumin levels < 32 g/L and ≥ 32 g/L, respectively. Survival analysis and log-rank testing showed that ccRCC patients with a post-operative serum albumin level < 32 g/L had a significantly decreased RFS (*P* = 0.00) and decreased OS (*P* = 0.00) than patients with a post-operative serum albumin level ≥ 32 g/L.

The type of surgical procedure is another important factor determining prognosis in patients with kidney cancer, especially in patients who undergo partial or radical nephrectomies. Of 329 patients, 224 and 105 underwent radical and partial nephrectomies, respectively. Log-rank testing showed that patients who underwent radical nephrectomies with post-operative serum albumin levels < 32 g/L had a significantly decreased RFS (*P* = 0.00) and decreased OS (*P* = 0.00) than patients with a post-operative serum albumin level ≥ 32 g/L. The RFS and OS among patients who underwent partial nephrectomies with a post-operative serum albumin level < 32 g/L were not significantly different compared with patients who had a post-operative serum albumin ≥32 g/L (*P* = 0.15 and 0.76, respectively).

## Discussion

Despite radical resection of kidney cancer, local recurrence or distant metastasis is commonplace [26–28]. With the exception of known prognostic factors, such as tumor stage, pathologic type, and type of surgical procedure, other potential prognostic factors warrant further elucidation [2, 3, 29, 30]. In this retrospective study we report for the first time that an early post-operative serum albumin level is another long-term prognostic factor after radical resection of kidney cancer.

To prevent confounding factors as much as possible, we excluded patients who had incomplete tumor resections, other life-threatening diseases, and adjuvant and/or neoadjuvant treatment. We excluded patients without serum protein and cell testing post-operatively, but it is unlikely that bias was introduced because no guidelines specify laboratory testing early after surgery without an indication. In our medical center, all radical surgeries for kidney cancer were performed by well-trained clinicians. To ensure a sufficient duration of follow-up, we only included patients who underwent surgery prior to May 2014. Approximately 42% of the enrolled patients had a duration of follow-up > 5 years and 94% of the enrolled patients had a duration of follow-up > 3 years. The median duration of follow-up was nearly 5 years, and no patients were lost to follow-up.

The serum albumin level nearly returned to the pre-operative level in all patients who had repeat serum protein testing. A decrease in the serum albumin level may primarily result from exudation into the extravascular space and attenuation by perioperative bleeding and fluid transfusion [11, 31]. We found a significant decrease in the hemoglobin concentration early after surgery coincident with the decrease in serum albumin level; however, the post-operative serum albumin level was significantly decreased compared with the serum albumin level regulated by the hemoglobin concentration (34.1 versus 37.0 g/L, *P* = 0.00), as follows: regulated serum albumin level ÷ pre-operative albumin level = post-operative hemoglobin concentration ÷ pre-operative hemoglobin concentration. This decrease is regarded as extravascular exudation. When the post-operative serum albumin level and extravascular exudation were entered into Cox regression, the results showed that the post-operative serum albumin level had a close association with RFS (HR = 0.85, *P* = 0.000) and OS (HR = 0.82, *P* = 0.000), unlike the association between extravascular exudation and RFS (HR = 1.00, *P* = 0.113) and OS (HR = 0.99, *P* = 0.116).

For patients who underwent pathologic-complete resection of kidney cancer, we entered other universally-accepted prognostic factors before survival analysis, including gender, age, nutritional status (BMI), tumor stage, pathologic type, Fuhrman grade, and type of surgical procedure. Survival analysis showed that patients who had an early post-operative serum albumin level < 32 g/L had a significantly decreased RFS and OS. Although patients in the group of patients with a lower post-operative albumin level were older, had a lower BMI, and more than stage III cancer and some laboratory values differed, multivariate Cox regression analysis admitted all of these factors, and the results showed that an early post-operative serum albumin level < 32 g/L was an independent risk factor for a decreased RFS and OS. Positive results were obtained, even when patients were confined to stages II and III. In contrast, the log rank test did not reveal significant differences in RFS (*P* = 0.360) and OS (*P* = 0.814) between stages II and III in our patients. Sub-group analysis showed an association between the post-operative serum albumin level and long-term prognosis based on patients with ccRC who underwent radical nephrectomies versus partial nephrectomies; however, only 26 patients with a post-operative serum albumin level < 32 g/L underwent partial nephrectomies. Indeed, the negative result may reflect the limited sample size.

Limited by the potential retrospective bias, the prognostic value of an early post-operative serum albumin level warrants more high-quality studies for further elucidation. Based on good category power (AUC = 0.71–0.8), an early post-operative serum albumin level deserves more attention in predicting prognosis after radical resection of kidney cancer, especially combined with other prognostic factors. The mechanisms underlying the association remain unclear. In addition, whether or not transfusion of albumin leads to decreased tumor recurrence and death after resection of kidney cancer should be investigated.

## Conclusion

In patients after curative resection of kidney cancer, this retrospective study revealed that an early post-operative serum albumin level < 32 g/L is an independent risk factor associated with a decreased RFS and decreased OS. Prospective trials and further research in additional patients are needed.

## Abbreviations

HR: Hazard Ratio
MSKCC: Memorial Sloan-Kettering Cancer Center
NCCN: National Comprehensive Cancer Network
OS: Overall Survival
RFS: Recurrence-free Survival
ROC: Receiver Operating Characteristic
